# TNFα sensitizes neuroblastoma cells to FasL-, cisplatin- and etoposide-induced cell death by NF-κB-mediated expression of Fas

**DOI:** 10.1186/s12943-015-0329-x

**Published:** 2015-03-19

**Authors:** Koen MO Galenkamp, Paulina Carriba, Jorge Urresti, Laura Planells-Ferrer, Elena Coccia, Joaquín Lopez-Soriano, Bruna Barneda-Zahonero, Rana S Moubarak, Miguel F Segura, Joan X Comella

**Affiliations:** Cell Signaling and Apoptosis Group, Fundacio Institut de Recerca de l’Hospital Universitari de la Vall d’Hebron, Edifici Collserola, Passeig Vall d’Hebron 119-129, 08035 Barcelona, Spain; Laboratory of Translational Research in Pediatric Cancer, Fundacio Institut de Recerca de l’Hospital Universitari de la Vall d’Hebron, Edifici Collserola, Passeig Vall d’Hebron 119-129, 08035 Barcelona, Spain

**Keywords:** Neuroblastoma, Fas (CD95/APO-1), TNFα, NF-κB, Cisplatin, Etoposide, Apoptosis

## Abstract

**Background:**

Patients with high-risk neuroblastoma (NBL) tumors have a high mortality rate. Consequently, there is an urgent need for the development of new treatments for this condition. Targeting death receptor signaling has been proposed as an alternative to standard chemo- and radio-therapies in various tumors. In NBL, this therapeutic strategy has been largely disregarded, possibly because ~50-70% of all human NBLs are characterized by caspase-8 silencing. However, the expression of caspase-8 is detected in a significant group of NBL patients, and they could therefore benefit from treatments that induce cell death through death receptor activation. Given that cytokines, such as TNFα, are able to upregulate Fas expression, we sought to address the therapeutic relevance of co-treatment with TNFα and FasL in NBL.

**Methods:**

For the purpose of the study we used a set of eight NBL cell lines. Here we explore the cell death induced by TNFα, FasL, cisplatin, and etoposide, or a combination thereof by Hoechst staining and calcein viability assay. Further assessment of the signaling pathways involved was performed by caspase activity assays and Western blot experiments. Characterization of Fas expression levels was achieved by qRT-PCR, cell surface biotinylation assays, and cytometry.

**Results:**

We have found that TNFα is able to increase FasL-induced cell death by a mechanism that involves the NF-κB-mediated induction of the Fas receptor. Moreover, TNFα sensitized NBL cells to DNA-damaging agents (i.e. cisplatin and etoposide) that induce the expression of FasL. Priming to FasL-, cisplatin-, and etoposide-induced cell death could only be achieved in NBLs that display TNFα-induced upregulation of Fas. Further analysis denotes that the high degree of heterogeneity between NBLs is also manifested in Fas expression and modulation thereof by TNFα.

**Conclusions:**

In summary, our findings reveal that TNFα sensitizes NBL cells to FasL-induced cell death by NF-κB-mediated upregulation of Fas and unveil a new mechanism through which TNFα enhances the efficacy of currently used NBL treatments, cisplatin and etoposide.

**Electronic supplementary material:**

The online version of this article (doi:10.1186/s12943-015-0329-x) contains supplementary material, which is available to authorized users.

## Background

Neuroblastoma (NBL) is a solid tumor that arises from neuronal crest cells of the sympathetic nervous system. The most common form of cancer in infancy, NBL causes 15% of cancer-related deaths in children. The tumors have remarkable heterogeneity, which become evident in the clinic where patients can show spontaneous regression or rapid and fatal tumor progression. Over the years, significant advances have been made in the treatment of low- and intermediate-risk patients, thus allowing reaching high survival rates; however, the 5-year survival rate of patients in the high-risk group is still below 50% [1-3].

High-risk NBLs are treated with surgery, chemotherapy, radiotherapy, and/or the use of biological agents. Most of the therapeutic strategies used in NBL interfere with cell cycle progression and DNA synthesis or function, thereby causing DNA damage and the induction of apoptosis through the intrinsic and extrinsic apoptotic pathways [4].

The extrinsic or Death receptor (DR) pathway is activated by cell surface receptors of the tumor necrosis factor receptor (TNFR) family, which includes receptors for TNFα, FasL, and TNF-related apoptosis-inducing ligand (TRAIL) [5-7]. These receptors contain a death domain in their cytosolic tail which upon receptor activation leads to context-dependent outcomes such as apoptosis, necroptosis, or pro-survival signaling. The targeting of DR signaling has been proposed and studied for the treatment of various types of cancers [8-10]. For NBL tumors, this strategy has been largely disregarded, possibly because caspase-8 silencing occurs in 50-70% of all human NBLs [11-14]. However, a significant group of NBL patients do express caspase-8 and could benefit from treatments that induce DR activation. Given that TNFα is able to upregulate Fas expression in human cancer cell lines and sensitize them to FasL-induced cell death [15-17], we sought to investigate whether TNFα and FasL combination could be therapeutically relevant in NBL.

We found that TNFα treatment primes a subset of NBLs for FasL-induced cell death by triggering the NF-κB-mediated upregulation of Fas. Moreover, TNFα pre-treatment increased cisplatin- and etoposide-induced caspase-8 cleavage and cell death in NBL cells that express both Fas and caspase-8. Our findings suggest that selected NBL patients could benefit from treatments that target TNFR1 and upregulate Fas expression.

## Results

### TNFα and FasL co-treatment induces cell death in SK-N-AS cells

**Figure 1:**
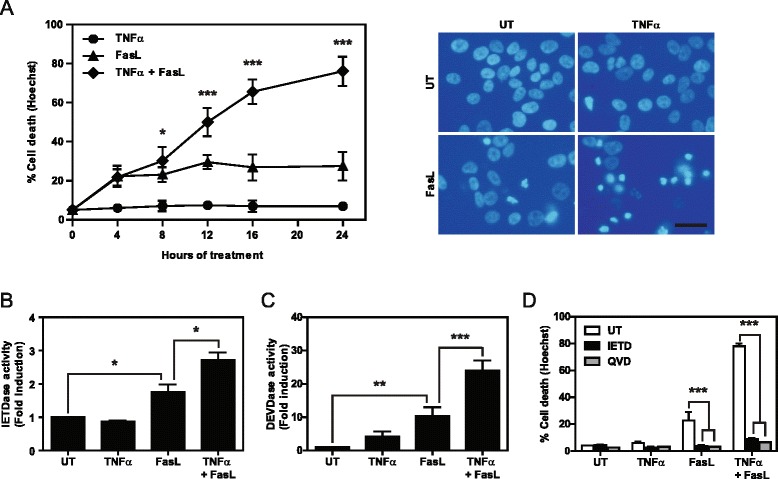
**Co-treatment with TNFα and FasL induces cell death in SK-N-AS cells. A**. SK-N-AS cells were left untreated (UT) or treated for the indicated times with 100 ng/ml Fc:hFasL, 100 ng/ml TNFα, or both. Cell death was assessed by Hoechst staining. Right images are representative of nuclear staining after 24 h of treatment. Scale bar, 20 μm. **B-C**. Caspase activity assay in SK-N-AS cells left untreated (UT) or treated for 8 h with 100 ng/ml TNFα, 100 ng/ml Fc:hFasL or both incubating with Z-IETD-Afc to measure caspase-8 **(B)** or Ac-DEVD-Afc to measure caspase 3/7 activity **(C)**. **D**. Cell death assay in SK-N-AS cells treated or not (UT) for 24 h with 100 ng/ml TNFα, 100 ng/ml Fc:hFasL, or both in the presence of the caspase-8 inhibitor (50 μM IETD) or the pan-caspase inhibitor (10 μM QVD). Cell death was assessed by Hoechst staining *p ≤ 0.05; **p ≤ 0.01; ***p ≤ 0.001.

To ascertain whether simultaneous treatment with FasL and TNFα induces cell death in NBL cells, we used the caspase-8-expressing NBL cell line SK-N-AS. TNFR1 was activated with soluble TNFα and the Fas receptor with trimeric Fc:hFasL [18-20]. Cell death assessment by Hoechst staining showed that TNFα or FasL treatment alone barely induced cell death (~5% and 20% respectively). However, the combination of the two cytokines caused an increase in cell death after 8 h of treatment, as compared to treatment with TNFα or FasL alone (Figure 1A). After 24 h, nearly all cells in the co-treatment condition were dead. Moreover, an increase in caspase-8 and caspase-3/7 activity was observed after simultaneous treatment with TNFα and FasL (Figure 1B-C). The use of the caspase-8 specific inhibitor IETD or the pan-caspase inhibitor QVD fully abrogated TNFα/FasL-induced cell death, thereby indicating that apoptosis triggered by FasL is the main mechanism of loss of viability (Figure 1D).

### TNFα primes SK-N-AS cells for FasL-induced cell death by upregulating Fas

**Figure 2:**
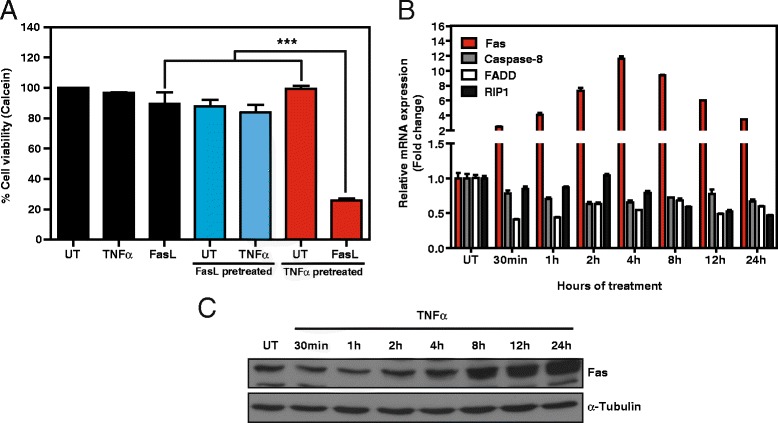
**TNFα primes for FasL-induced cell death and upregulates Fas expression. A**. SK-N-AS cells were left untreated or were pre-treated for 24 h with 100 ng/ml TNFα or 100 ng/ml Fc:hFasL and subsequently washed and left untreated (UT) or treated for 24 h with 100 ng/ml TNFα or 100 ng/ml Fc:hFasL. Cell viability was assessed by calcein AM staining. **B**. SK-N-AS cells were treated or not with 100 ng/ml TNFα for the indicated times and mRNA levels of the indicated genes were assessed by qRT-PCR. mRNA expression levels were normalized using 18S. **C**. Western blot of Fas expression in SK-N-AS cells treated with 100 ng/ml TNFα for the indicated times. α-Tubulin was used as loading control. ***p ≤ 0.001.

Next, we analyzed whether the phenotypic effects of the TNFα/FasL co-treatment were caused by the FasL and TNFα receptors signaling in synergy or whether one DR was sensitizing for apoptotic signaling by the other DR. Sequential treatment with TNFα or FasL followed by FasL or TNFα administration, respectively, revealed that TNFα was able to sensitize SK-N-AS cells to FasL-induced cell death (Figure 2A). On the contrary, FasL pre-treatment did not sensitize the cells to TNFα. The increase in caspase-8 activity during the co-treatment and the abrogation of cell death when using the caspase-8 inhibitor IETD suggested that TNFα induces molecular changes upstream of caspase-8 activation. Therefore, to address this point, we characterized the effects of TNFα on the expression of various components of the DISC complex (Figure 2B). Interestingly, a significant increase was observed only in Fas mRNA levels whereas minimal variations were detected for caspase-8, FADD, and RIP1 (Figure 2B). The increase in Fas mRNA levels was confirmed at the protein level by Western blot as early as 8 h after TNFα treatment (Figure 2C), which is consistent with the increase in cell death observed after simultaneous treatment with TNFα and FasL.

### Newly synthesized Fas is exposed to the plasma membrane and favors DISC formation

**Figure 3:**
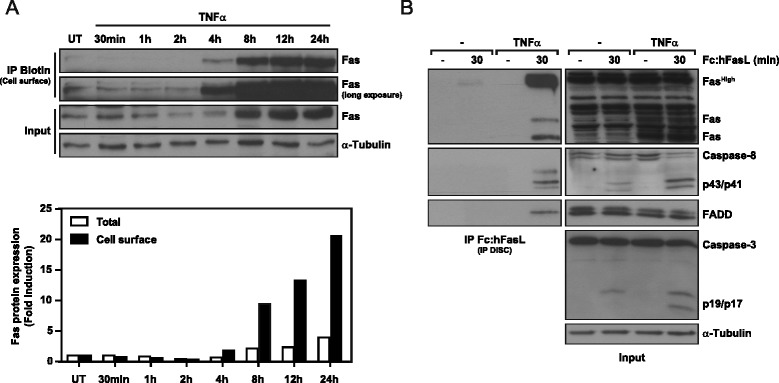
**TNFα-induced Fas is exposed to the cell surface and enhances FasL-induced DISC formation. A**. SK-N-AS cells were treated with 100 ng/ml TNFα for the indicated times, and cell surface levels of Fas protein were assessed by cell surface biotinylation and subsequent Western blot. Below, quantification of total and cell surface Fas expression normalized vs. matching α-Tubulin controls. **B**. DISC (Death inducing signaling complex) formation assay in SK-N-AS cells pre-treated or not with 100 ng/ml TNFα and treated for 30 min with 2.5 μg/ml Fc:hFasL. The DISC proteins were immunoprecipitated by pulling down Fc:hFasL with protein G-Sepharose and analyzed by Western blot.

The increase in FasL-mediated cell death could be explained by exposure of the newly synthesized Fas receptor to the cell surface. To test this notion, we used a cell surface biotinylation assay to analyze the cellular distribution of Fas after TNFα treatment. An increase in total and cell surface Fas was already observed after 4 h of TNFα treatment, peaking at 12 h, after which the expression was maintained for at least another 12 h (Figure 3A). To further confirm the functionality of the newly synthesized Fas, a DISC formation assay was performed before and after TNFα pre-treatment. Indeed, FasL co-immunoprecipitated with FADD and caspase-8 only in cells pre-treated with TNFα (Figure 3B). Interestingly, in the absence of this pre-treatment, we were only able to immunoprecipitate low levels of high molecular weight Fas (~150 kDa) and in these conditions we did not detect caspase-8 or FADD immunoprecipitation. The analysis of input cell lysates confirmed that caspase-8 and −3 were cleaved only after FasL treatment and could be increased by TNFα pre-treatment (Figure 3B). These data demonstrate that cell surface exposure of Fas shows similar kinetics as the newly synthesized Fas, thereby suggesting a rapid translocation of newly synthesized Fas to the surface. Furthermore, the increased cell surface expression of Fas enhanced FasL-induced DISC formation, which led to the activation of the extrinsic apoptotic pathway.

### TNFα-induced Fas is transcriptionally regulated by NF-κB

**Figure 4:**
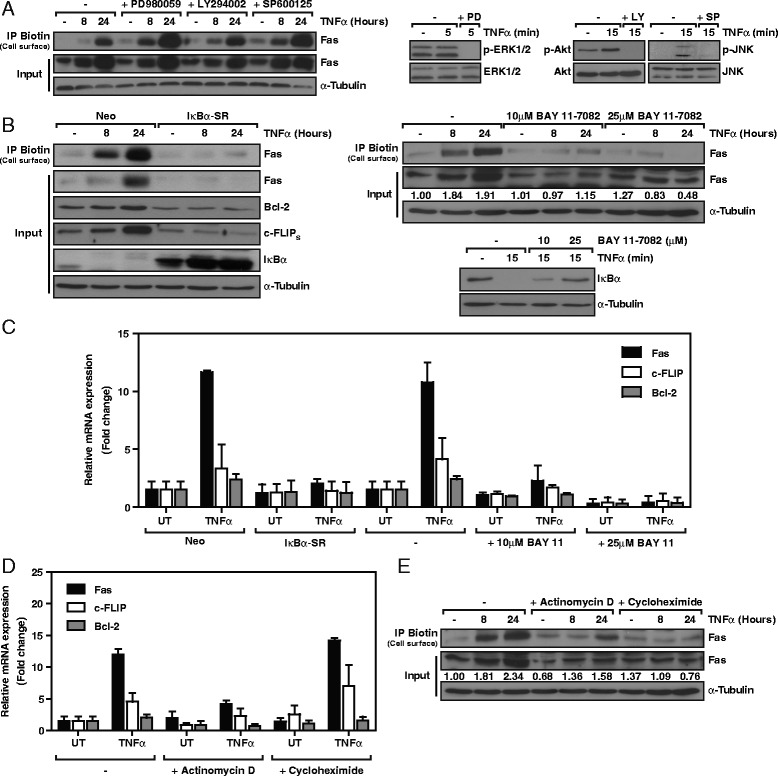
**NF-κB mediates TNFα-induced Fas expression and exposure to the cell surface. A**. Left panel, SK-N-AS cells were treated with 25 μM PD980059, 20 μM LY294002 or 20 μM SP600125 prior to treatment with 100 ng/ml TNFα. At the indicated times, cell surface proteins were biotinylated, isolated, and analyzed by Western blot. Right panel, phospho-protein levels assessed by Western blot to confirm efficacy of PD980059 (PD), LY294002 (LY), and SP600125 (SP). **B**. Left panel, control (Neo) and SuperRepressor/SR-IκBα-infected SK-N-AS cells were left untreated (−) or treated with 100 ng/ml TNFα for 8 h and 24 h. Cell surface proteins were biotinylated, isolated, and analyzed by Western blot. Right panel, analysis of cell surface and total Fas levels. SK-N-AS cells were incubated with 10 μM and 25 μM of the NF-κB inhibitor BAY 11–7082 prior treatment with 100 ng/ml TNFα. Below, quantification of total Fas expression normalized vs. matching α-Tubulin controls. IκBα degradation Western blot confirmed the efficacy of BAY 11–7082. **C**. Control (Neo) and SuperRepressor/SR-IκBα-infected or control (−) and BAY 11–7082 pre-treated SK-N-AS cells were left untreated (UT) or treated with 100 ng/ml TNFα. mRNA levels of the indicated genes were assessed after 4 h by qRT-PCR. **D**. qRT-PCR assessment of the indicated genes, 4 h after 100 ng/ml TNFα treatment of SK-N-AS cells in combination with 20 nM actinomycin D or 1 μg/ml cycloheximide. **E**. Cell surface biotinylation assay in SK-N-AS cells pre-treated with 20 nM actinomycin D or 1 μg/ml cycloheximide and treated with 100 ng/ml TNFα for the indicated times. Protein levels were analyzed by Western blot. Below, quantification of total Fas expression normalized vs. matching α-Tubulin controls. All conditions were pre-incubated with 10 μM of the caspase inhibitor QVD to avoid cell death-related effects.

TNFα has been shown to induce gene expression by activating various signaling pathways, such as those of ERK1/2, PI3K, and JNK kinases and transcription factors like NF-κB [5,6,21]. Therefore, we proceeded to analyze Fas expression after TNFα treatment combined with specific inhibitors: PD98059 (ERK1/2), LY294002 (PI3K), SP600125 (JNK), BAY 11–7082 (NF-κB), or overexpressing Super Repressor (SR), a mutated form of the NF-κB inhibitor IκBα that inhibits NF-κB signaling [22,23]. While the inhibition of ERK1/2, PI3K or JNK did not block the upregulation of Fas by TNFα (Figure 4A), the inhibition of the NF-κB -pathway by overexpression of SR or treatment with BAY 11–7082 did fully abrogate the upregulation of Fas induced by TNFα at the protein level (Figure 4B). Other known NF-κB targets, such as Bcl-2 [24] and c-FLIP_S_ [25], were used as controls. Moreover, the use of SR overexpression or treatment with BAY 11–7082 blocked the TNFα-induced upregulation of Fas mRNA or known NF-κB targets such as c-FLIP and Bcl-2 (Figure 4C). Further assessment of TNFα-induced gene transcription and mRNA translation was performed with the use of DNA transcription (Actinomycin D) and mRNA translation (Cycloheximide) inhibitors (Figure 4D-E). Both inhibitors blocked the upregulation of Fas protein levels induced by TNFα, whereas only actinomycin D was able to inhibit TNFα-induced upregulation of Fas mRNA. Cycloheximide did not result in abrogation of Fas mRNA induction, thereby confirming the direct NF-κB -mediated transcriptional regulation of this protein (Figure 4D-E). In summary, our results reveal that the upregulation of Fas mRNA occurs rapidly after TNFα treatment through activation of the NF-κB -mediated transcription of the Fas gene. Moreover, the inhibition of Fas upregulation by the overexpression of SR, treatment with BAY 11–7082 (Figure 4B), or by blocking its synthesis with actinomycin D and cycloheximide treatment (Figure 4E) prevented the cell surface exposure of Fas.

### TNFα primes for cisplatin- and etoposide-induced activation of caspase-8 and cell death

**Figure 5:**
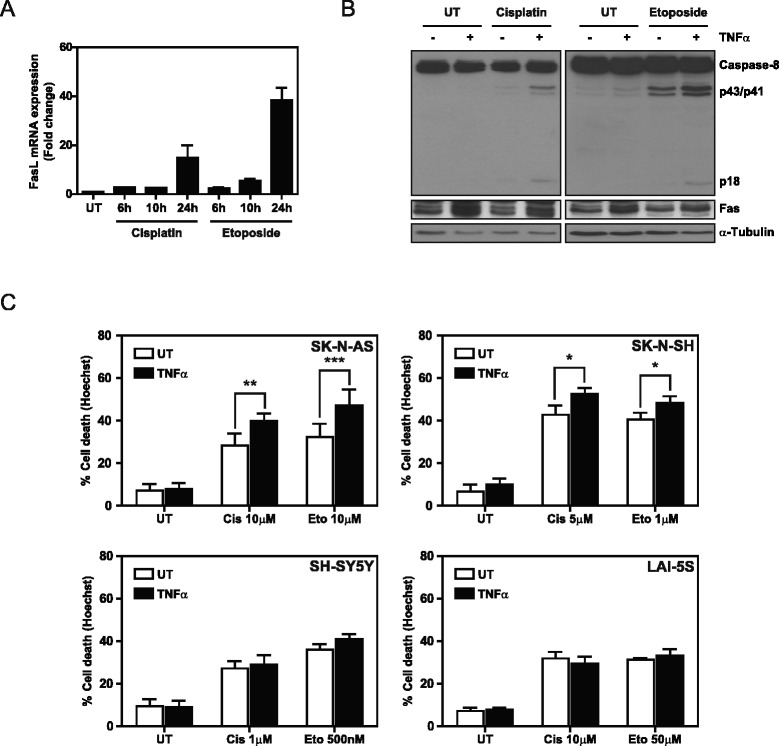
**TNFα is able to sensitize NBL cell lines to cisplatin- and etoposide-induced cell death. A**. FasL mRNA levels were assessed by qRT-PCR in cisplatin- (30 μM) and etoposide- (30 μM) treated SK-N-AS cells for the indicated times. mRNA levels were normalized using 18S mRNA. **B**. Caspase-8 cleavage was analyzed by Western blot in SK-N-AS cells pre-treated with 100 ng/ml TNFα for 24 h after cisplatin (30 μM) and etoposide (30 μM) treatments. **C**. Cell death assay by Hoechst staining in the indicated cell lines pre-treated with 100 ng/ml TNFα for 24 h and treated with the indicated concentrations of cisplatin (Cis) and etoposide (Eto) for 48 h. *p ≤ 0.05; **p ≤ 0.01; *** p ≤ 0.001.

The FasL/Fas system has been shown to participate in cell death mechanisms triggered by DNA-damaging agents currently used in NBL therapy such as cisplatin and etoposide [26,27]. Therefore, we addressed whether TNFα treatment enhances the cytotoxic effect of these two drugs. Cisplatin and etoposide have been shown to induce FasL expression in NBL cells [27]. We confirmed these observations in SK-N-AS cells, as an increase in FasL mRNA was detected after 24 h of etoposide or cisplatin treatment (Figure 5A). Furthermore, the induction of FasL by treatment with these chemotherapeutic agents concurred with the activation of caspase-8, as reflected by caspase-8 cleavage (Figure 5B). Indeed, TNFα treatment upregulated Fas expression in SK-N-AS cells and enhanced the cleavage of caspase-8 induced by cisplatin (Figure 5B left) and by etoposide (Figure 5B right). We next analyzed the functional consequences of these observations in a subset of NBL cell lines (SK-N-AS, SK-N-SH, SH-SY5Y, and LAI-5S) and assessed cell death by Hoechst staining (Figure 5C). Notably, only the SK-N-AS and SK-N-SH cell lines showed an increase in cisplatin- and etoposide-induced cell death when primed with TNFα, whereas no changes in cell death were observed for the SH-SY5Y and LAI-5S cell lines.

### NBLs show heterogeneity in TNFα-induced Fas expression, thereby explaining the priming for cisplatin- and etoposide-induced cell death

**Figure 6:**
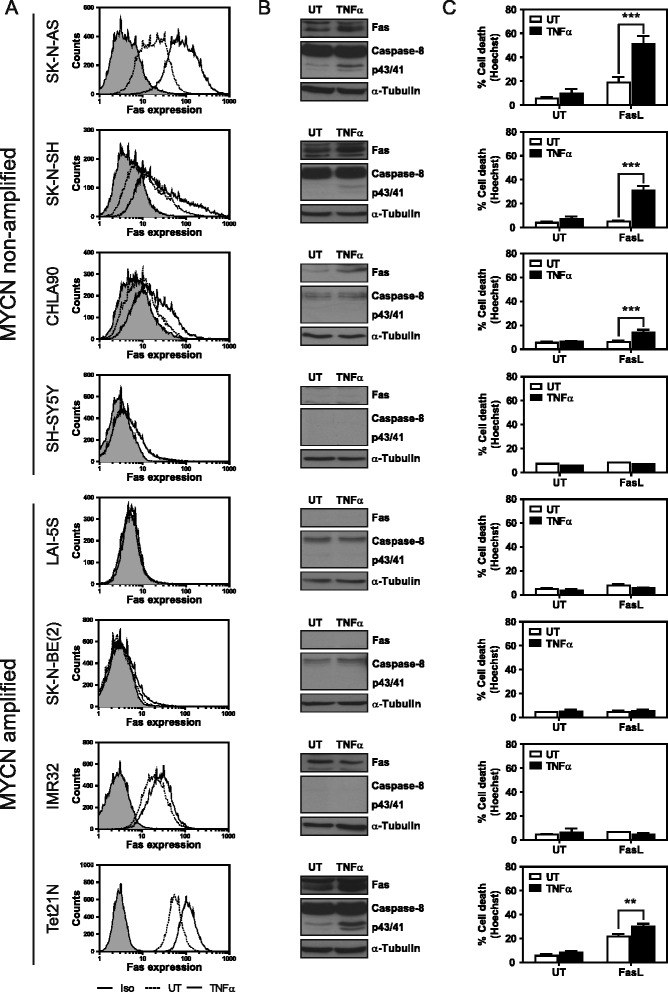
**NBLs show heterogeneous Fas expression in response to TNFα treatment. A**. NBL cell lines were treated with 100 ng/ml TNFα or left untreated (UT) for 24 h. Fas expression was analyzed by cytometry using a PE-conjugated Fas or isotype antibody. **B**. Cells were treated for 24 h with 100 ng/ml TNFα or were left untreated (UT). Protein expression levels were analyzed by Western blot. **C**. Cell death assay by Hoechst staining in the indicated cell lines pre-treated with 100 ng/ml TNFα for 24 h and treated for another 24 h with 100 ng/ml Fc:hFasL, or 1 ng/ml Fc:hFasL for Tet21N cells. **p ≤ 0.01; ***p ≤ 0.001.

NBLs are known to have a high degree of heterogeneity [1-3], which may explain why some NBL cell lines are not primed by TNFα for cisplatin- and etoposide-induced cell death. Therefore, we assessed the expression of Fas and its modulation by TNFα treatment in a set of eight NBL cell lines. In addition to the SK-N-AS cell line, TNFα upregulated Fas expression in the SK-N-SH, CHLA90, and Tet21N cell lines, as observed by flow cytometry (Figure 6A) and Western blot (Figure 6B). Furthermore, TNFα was also able to sensitize these cells to FasL-induced apoptosis, as determined by Hoechst staining (Figure 6C) and caspase-3/7 activity (Additional file 1: Figure S1). In contrast, the SH-SY5Y, LAI-5S, IMR32, and SK-N-BE(2) cell lines did not show changes in Fas expression (Figure 6A-B), FasL-induced cell death (Figure 6C), or caspase-3/7 activity (Additional file 1: Figure S1) after TNFα treatment. Interestingly, these cells showed TNFα-induced IκBα degradation and the upregulation of other known NF-κB -target genes, such as Bcl-2 [24] and/or cIAP2 [28] (Additional file 2: Figure S2A-B).

A correlation could be observed between the expression of both Fas and caspase-8, the induction of cell death by FasL and the sensitization thereof by TNFα. Cells that only express one of the two proteins did not show FasL-induced cell death and could not be sensitized by TNFα. However, cells that express both proteins, Fas and caspase-8, did show FasL-induced cell death and TNFα-induced sensitization. Furthermore, the levels of Fas and caspase-8 expression concur with the FasL-induced cell death response. CHLA90 cells, with low levels of Fas and caspase-8, exhibit little FasL-induced cell death when compared to Tet21N cells, which show high levels of Fas and caspase-8 expression. Due to these high levels and the corresponding cell death response, Tet21N cells had to be treated with lower levels of FasL for the TNFα-induced sensitization to become apparent, since 100 ng/ml FasL induced a near complete cell death response (data no shown).

These findings demonstrate that although NF-κB is activated and induces gene transcription in all the NBL cell lines studied here, there is a subset of NBL cell lines in which Fas expression is not upregulated in response to TNFα treatment. These observations concur with our previous observations in which we determined that these cells were primed neither for cisplatin- nor etoposide-induced cell death when targeted with TNFα.

### Interferon-γ reconstitutes caspase-8, upregulates Fas expression, and primes NBL for FasL-induced cell death

**Figure 7:**
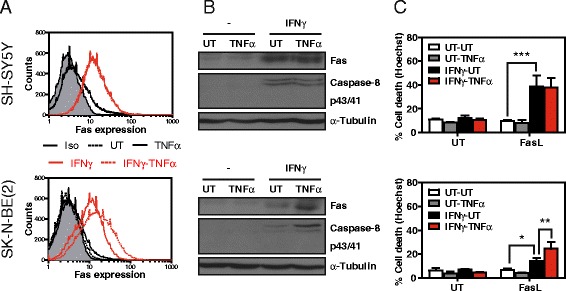
**IFNγ renders caspase-8- and Fas-deficient NBLs sensitive to FasL. A**. The indicated cell lines were treated with 100 ng/ml interferon-γ (IFNγ) or were left untreated (UT) for 24 h. Cells were then treated or not for 24 h with 100 ng/ml TNFα and Fas expression was analyzed by cytometry using a PE-conjugated Fas or an isotype antibody. **B**. Fas and caspase-8 expression analysis by Western blot in the indicated cell lines pre-treated for 24 h with 100 ng/ml interferon-γ (IFNγ) and treated with 100 ng/ml TNFα for 24 h. **C**. Cell death assay in NBL cell lines pre-treated or not (UT) with 100 ng/ml IFNγ for 24 h and thereafter treated or not with 100 ng/ml TNFα for 24 h. Next, cells were treated for an additional 24 h with 100 ng/ml Fc:hFasL. *p ≤ 0.05; **p ≤ 0.01; ***p ≤ 0.001.

Interferon-γ (IFNγ) is known to render NBL cells sensitive to FasL-induced cell death by reconstituting caspase-8 and upregulating Fas expression [29-31]. Here we studied whether NBL treatment with IFNγ promotes TNFα-induced Fas expression in cell lines that previously did not show Fas induction in response to TNFα treatment (i.e. SH-SY5Y and SK-N-BE(2)). We confirmed that IFNγ upregulates caspase-8 and Fas expression in these cell lines (Figure 7A-B). The SK-N-BE(2) cell line showed a TNFα-induced increase in caspase-8 and Fas expression after IFNγ treatment. However, in the SH-SY5Y cells, TNFα did not modulate the expression of either protein. According to these observations, IFNγ sensitized SK-N-BE(2) and SH-SY5Y cells to FasL-induced cell death (Figure 7C) and caspase 3/7 activity (Additional file 1: Figure S1). For the SK-N-BE(2) cells, subsequent TNFα treatment further increased sensitization to FasL-induced cell death. In contrast, SH-SY5Y cells, which did not show a further increase in caspase-8 or Fas levels, did not show further sensitization to FasL-induced cell death after stimulation with TNFα. These data indicate that the induction of Fas expression by TNFα in NBLs cannot always be recovered by IFNγ treatment, thus pointing to different levels of Fas regulation.

## Discussion

Many patients with high-risk NBL tumors continue to have a poor prognosis. Consequently, ongoing efforts are being channeled into the development of new treatments or the discovery of therapeutic agents that can increase the efficacy of current clinical regimes—cisplatin and etoposide being examples of such drugs [2]. Here we describe that the activation of TNFR1 increases susceptibility to FasL-, cisplatin- and etoposide-induced cell death through the NF-κB -mediated upregulation of Fas, a target that has received little attention for NBL therapies. The newly synthesized Fas is exposed to the cell surface and incorporated into the DISC complex upon ligand binding, thereby triggering the activation of caspases and inducing apoptotic cell death.

Soluble TNFα exerts its effects through the binding and activation of the ubiquitously expressed TNFR1 receptor [5-7,18,19]. Depending on the cellular context, TNFα stimulation induces apoptosis, necroptosis, or pro-survival signaling through the activation of caspases, kinases, and transcription factors such as NF-κB [5-7,21]. For NF-κB activation, TNFR1 binds the adaptor protein TRADD through interaction with its death domain. This interaction allows the recruitment of the adapter protein RIP1 and the E3 ligases TRAF2/5 and cIAP1/2, thereby inducing the ubiquitination of RIP1. This shapes the platform for recruitment and activation of the IKK complex that induces phosphorylation of the cytoplasmic NF-κB inhibitor IκBα, thereby targeting it for ubiquitination and subsequent proteasomal degradation. Degradation of IκBα mediates the release of NF-κB and allows its translocation to the nucleus where it can induce gene transcription. According to our data and data from others, *FAS* is amongst the genes that can be induced by NF-κB. Chan *et al.* and Liu *et al*. have previously identified the p65/RelA binding site in the Fas promoter and confirmed TNFα-induced NF-κB -mediated upregulation of Fas [32,33]. Here, we were able to demonstrate the NF-κB -mediated regulation in NBLs and discarded regulation of Fas expression by other pathways known to be activated by TNFR1 (i.e. ERK1/2, PI3K, and JNK).

Given the participation of the Fas/FasL system in the mechanisms of cell death caused by DNA-damaging agents such as cisplatin and etoposide [26,27], we studied the possibility of improving the efficacy of these drugs by combined treatment with TNFα. Our results showed that TNFα pre-treatment increased cisplatin- and etoposide-induced cell death in two of the four NBL cell lines studied. Similarly, Benedetti *et al.* reported that TNFα acts in synergy with cisplatin in renal proximal tubular cells, inducing an increase in cell death by prolonging JNK activation and inhibiting NF-κB translocation to the nucleus [34,35]. However, our data indicate that the TNFα-induced priming for cisplatin- and etoposide-induced cell death depends on NF-κB -mediated induction of Fas expression and caspase-8 cleavage.

Remarkably, not all the NBL cell lines studied were primed by TNFα for cisplatin- and etoposide-induced cell death. To predict the benefit of the TNFα combination therapy, we analyzed the expression of Fas and the modulation thereof by TNFα in a set of eight NBL cell lines. In four of the eight NBL cell lines, TNFα upregulated Fas expression. Furthermore, we observed that only the cell lines that showed TNFα-induced upregulation of Fas expression also displayed TNFα-induced priming to FasL-, cisplatin-, and etoposide-induced cell death. The cell lines that showed TNFα-induced priming also displayed Fas and caspase-8 expression, whereas cell lines that were not primed by TNFα showed the expression of only one of the two proteins. The response to TNFα treatment was not related to other frequent NBL alterations, such as MYCN amplification or p53 functional status (see Table 1).

**Table 1:** **Neuroblastoma characteristics and their modulation by TNFα**

		**MYCN non-amplified** **[** 36 **,** 37 **]**				**MYCN amplified** **[** 36 **,** 37 **]**			
		**SK-N-AS**	**SK-N-SH**	**CHLA90**	**SH-SY5Y**	**LAI-5S**	**SK-N-BE(2)**	**IMR32**	**Tet21N**
**Expression**	**Fas**	+	+	+/−	+/−	-	-	+	++
	**Caspase-8**	++	++	+/−	-	+/−	+/−	-	++
	**p53**	N [38]	F [39]	N [40]	F [39]	N [41]	N [40]	F [39]	F [39]
**TNFα-induced**	**Fas**	++	+	+	-	-	-	-	++
	**Sensitization to etoposide/cisplatin**	++	+	NA	-	-	NA	NA	NA
**FasL-induced cell death**	**UT**	+	+/−	-	-	-	-	-	++
	**TNFα**	+++	+++	+	-	-	-	-	+++

*Abbreviations: F* Functional, *N* Non-functional, *NA* Not available.

The mechanism by which Fas is silenced in NBL and why some cell lines do not respond to the TNFα-induced Fas regulation remains to be clarified. In the NBL cell lines addressed, we confirmed NF-κB activation after TNFα treatment and detected the induction of other known NF-κB target genes, such as cIAP2 and Bcl-2 [24,28]. One possible mechanism to explain this lack of Fas induction is that TNFα treatment stimulates the formation of different NF-κB heterodimers or NF-κB was post-transcriptionally modified, which may drive specific gene expression [42]. An alternative mechanism to account for the incapacity of TNFα to induce Fas expression can be found at the level of epigenetic regulation of the Fas gene. Methylation of the Fas promoter has been reported in various types of tumors, including NBL [43-45]. IFNγ has been shown to restore caspase-8 and Fas expression in NBL cells [29-31,46,47] and to render them sensitive to FasL treatment. Consequently, IFNγ may also prime caspase-8- or Fas-deficient NBL cells for the TNFα combination therapy. Indeed, we confirmed that IFNγ primes these NBL cells for FasL-induced cell death. However, IFNγ treatment did not sensitize all the NBL cell lines to the TNFα-induced upregulation of Fas. These findings suggest that the expression of Fas in NBLs is regulated at various levels and that it differs between NBLs.

Recent studies have described the benefits of TNFα in combination with doxorubicin [48] or melphalan [49] for the treatment of solid tumors. Due to its low toleration in systemic treatment, various TNFα fusion proteins have been developed for localized treatment [50], some of which show promise and have entered clinical trials [49,51,52]. These findings break ground for the use of TNFα in the treatment of NBL in combination with cisplatin and etoposide.

Our results suggest that NF-κB -mediated upregulation of Fas by TNFα could be a new approach for the treatment of NBL patients. These findings are in contradiction to the current dogma in which NF-κB inhibition is seen as a strategy for cancer treatment, since NF-κB has been implicated in promoting cancer initiation, development, and metastasis [53,54]. NF-κB activation is known to promote cell survival by upregulating anti-apoptotic proteins, such as Bcl-2, c-FLIP, and cIAP2 thereby inhibiting DR-induced apoptosis [24,25,28]. However, NF-κB is also able to promote apoptosis through the induction of pro-apoptotic proteins, such as Fas [32,33], Bax [55], DR5 [56], and DR6 [57]. Our study supports the evidence that NF-κB triggers pro-apoptotic signaling in a subset of NBL cells through Fas upregulation, which tips the scale towards apoptotic cell death.

## Conclusions

The results of this study contribute to our understanding of Fas expression, its regulation by TNFα in a NBL setting, and its implications in the treatment of NBL tumors. Although TNFα is mostly known for its pro-survival signaling [24,25,28], our results indicate that this cytokine has the capacity to prime caspase-8- and Fas-expressing NBLs for cisplatin- and etoposide-induced cell death. These findings pave the way for a new approach to improve clinical response to current NBL treatments.

## Methods

### Reagents

Unless stated otherwise, all biochemical reagents were purchased from Sigma-Aldrich (St. Louis, MO, USA). Recombinant Fc:hFasL was a generous gift of Dr. Pascal Schneider (University of Lausanne, Epalinges, Switzerland). Recombinant human TNF*α* and IFNγ were supplied by Biotrend (Köln, Germany). PD98059, SP600125, BAY 11–7082, Z-IETD-FMK, and Q-VD-OPH were purchased from Merck Millipore (Billerica, MA, USA).

### Cell culture

The human NBL cell lines SK-N-AS, LAI-5S, IMR32, SK-N-BE(2), and SH-SY5Y and the renal epithelial cell line HEK293T were cultured in DMEM (Thermo Fisher Scientific, Waltham, MA, USA) supplemented with 10% or 15% (SH-SY5Y) heat-inactivated FBS (FBSi, Thermo Fisher Scientific). The NBL cell lines SK-N-SH and CHLA90 were cultured in IMDM (Thermo Fisher Scientific) supplemented with 20% FBSi. The NBL cell line Tet21N was maintained in RPMI 1640 (Thermo Fisher Scientific) supplemented with 10% FBSi, 25 mM HEPES (Thermo Fisher Scientific), 200 μg/ml geneticin (G418), 0.5 μg/ml amphotericin B, and 10 μg/ml hygromycin B. Cell culture media was supplemented with 100U/ml penicillin and 100 μg/ml streptomycin (Thermo Fisher Scientific). Cultures were maintained at 37°C in a saturated atmosphere of 95% air and 5% CO_2_. CHLA90 cells were acquired from the Children’s Oncology Group Cell Line repository. SK-N-BE(2) and LAI-5S cells were from the Public Health England Culture Collections (Salisbury, UK). Tet21N cells were a kind gift from Dr. Manfred Schwab (DKFZ, Heidelberg, Germany). All other cell lines were acquired from the American Type Tissue Collection (ATCC, Manassas, VA, USA).

### Hoechst staining

After the indicated treatments, cells were fixed with 2% paraformaldehyde, permeabilized with 0.1% Triton™ X-100, and stained with 0.05 μg/ml Hoechst 33342. Cell death was assessed by counting viable and dead cells, by discriminating condensed and fragmented nuclei (apoptotic nuclear morphology type II), as described by Yuste *et al.* [58]. Quantification was performed in blind testing, and at least 500 cells were counted per condition.

### Caspase activity

After the indicated treatments, cells were harvested, washed with ice-cold PBS, lysed in caspase activity buffer (20 mM HEPES-NaOH, pH7.2, 10% sucrose, 150 mM NaCl, 5 mM EDTA, 1% Igepal CA-630, 0.1% CHAPS, and 1× EDTA-free Complete protease inhibitor mixture), and insoluble fractions were removed by centrifugation. The protein concentration of the lysate was quantified using the Lowry-based DC protein assay (Biorad, Hercules, CA, USA). Next, caspase activity was assessed by incubating 10 μg protein at 37°C in caspase activity buffer supplemented with 10 mM DTT and 50 μM of the fluorogenic substrate Z-IETD-Afc for caspase-8 activity or Ac-DEVD-Afc for caspase-3/7 activity (Merck Millipore). Caspase activity was assessed in a fluorometer using excitation and emission wavelengths of 405 nm and 535 nm, respectively.

### Calcein AM

After the indicated treatments, cells were incubated for 1 h at 37°C with 1 μM Calcein AM (Merck Millipore) diluted in DPBS (Thermo Fisher Scientific). Fluorescence was then assessed in a fluorometer using excitation and emission wavelengths of 485 nm and 535 nm, respectively.

### qRT-PCR

After treatment, cells were harvested, washed with ice-cold PBS, and RNA was isolated using the RNeasy Mini kit (Qiagen, Hilden, Germany) following the manufacturer’s instructions. Next, the RNA was retrotranscribed to cDNA using the High Capacity RNA-to-cDNA™ Kit (Thermo Fisher Scientific) and subjected to PCR analysis using Taqman® probes and Universal PCR Master Mix (Thermo Fisher Scientific). Taqman® probes: Fas (Hs00531110_m1), Caspase-8 (Hs01018151_m1), FADD (Hs00538709_m1), RIP1 (Hs00169407_m1), FasL (Hs00181225_m1), c-FLIP (Hs01116280_m1), Bcl-2 (Hs00608023_m1), and 18S (Hs03928990_g1).

### Cell surface biotinylation

Cell surface proteins were biotinylated, isolated, and collected by using the Pierce® Cell Surface Protein Isolation Kit (Thermo Fisher Scientific), following the manufacturer’s instructions, with the only exception of equalizing protein quantity and concentration before immunoprecipitation. Protein levels were determined by Western blot.

### DISC immunoprecipitation

For Fas DISC analysis, cells were treated with Fc:hFasL (2.5 μg/ml) for 30 min. The cells were then washed with ice-cold PBS, harvested, and lysed in ice-cold Triton lysis buffer (NaCl 150 mM, EDTA 10 mM, Tris–HCl pH7.4 10 mM, 1% Triton™ X-100, 1x EDTA-free complete protease inhibitor cocktail (Roche, Basel, Switzerland)). After lysate clearance by centrifugation, Fc:hFasL was immunoprecipitated from the supernatant by incubation with protein G-Sepharose beads for 1 h on an orbital shaker at 4°C. Next, the beads were washed 5x with ice-cold Triton lysis buffer, and the immunocomplexes were collected with elution buffer (Citrate 0.1 M, pH2.5). The pH was adjusted by adding 1/6 neutralizing buffer (Tris HCl 1 M, pH8.5). Protein levels were determined by Western blot.

### Western blot

Cells were harvested, washed with ice-cold PBS, and lysed in ice-cold Triton lysis buffer or boiling SET buffer (Tris–HCl pH7.4 10 mM, EDTA 1 mM, NaCl 150 mM, 1% SDS). Insoluble fractions were removed by centrifugation, and protein concentration of the supernatant was quantified. The cell lysates obtained (25 μg of protein) were resolved in SDS-polyacrylamide gels. Next, proteins were transferred onto PVDF Immobilon-P membranes (Merck Millipore) by electrophoresis. Membranes were blocked with 5% non-fat dry milk in 1× TBS and 0.1% Tween-20 and probed with the appropriate primary antibodies [anti-Fas (C-20), anti-FADD (S-18), anti-c-FLIP_S/L_(H-202), anti-IκBα (C-21), anti-cIAP2 (H-85) (Santa Cruz, Biotechnology, Santa Cruz, CA, USA), anti-α-Tubulin (Sigma-Aldrich), anti-Bcl-2 (Dako, Agilent Technologies, Santa Clara, CA, USA), anti-Caspase-3 and anti-Caspase-8 (Cell Signaling Technologies, Beverly, MA, USA)] and the corresponding peroxidase-conjugated secondary antibodies (Sigma-Aldrich).

### Plasmids

The Super-repressor IκBα (SR) cDNA was subcloned from the validated pcDNA3 expression vector [22,59] into the lentiviral pWPI expression vector. SR was expressed under the control of the constitutively active EF-1 Alpha promoter.

### Lentiviral production and cell infection

Lentiviruses were produced in HEK293T cells by Lipofectamine 2000 (Thermo Fisher Scientific) co-transfection of pWPI-derived constructs, pSPAX2, and pM2G in a 3:2:1 ratio, respectively. Cells were allowed to generate lentiviruses for 48 h, after which the lentivirus-bearing medium was collected and passed through a Whatman® 45 μm filter (GE Healthcare, Little Chalfont, UK). For infection, the lentivirus-bearing medium was added to the host cells in combination with 8 μg/ml polybrene. Infection efficiency was assessed by direct counting of GFP-positive cells, and infection was repeated until an efficiency of ≥95% was reached.

### Flow cytometry

After the indicated treatments, cells were detached with cell dissociation buffer (PBS, 5 mM EDTA), harvested, washed 2× with ice-cold PBS and 1× with ice-cold FACS buffer (PBS, 2% FBSi, 0.02% sodium azide), and then incubated for 30 min on ice with a PE-conjugated monoclonal antibody against Fas or its matched isotype (Becton Dickinson, Franklin Lakes, NJ, USA). Thereafter, cells were washed 2× and resuspended in ice-cold FACS buffer. Fas expression was assessed by a FACSCalibur™ flow cytometer (Becton Dickinson).

### Statistical analysis

All the experiments were repeated at least three times. Values are expressed as mean ± SD. Statistical significance was determined by one-way or two-way ANOVA using GraphPad Prism v5 (GraphPad Software, La Jolla, CA, USA).

## Additional files

Additional file 1: Figure S1. TNFα pre-treatment sensitizes a subset of NBLs to FasL-induced caspase-3/7 activity. The indicated cell lines were pre-treated or not for 24 h with 100 ng/ml TNFα and were left untreated (UT) or treated for 4 h with 100 ng/ml Fc:hFasL, or 1 ng/ml Fc:hFasL for Tet21N cells. Before Fc:hFasL treatment, SH-SY5Y and SK-N-BE(2) cells were treated for 24 h with 100 ng/ml interferon-γ (IFNγ) or not (UT), and an additional 24 h with 100 ng/ml TNFα or were left untreated (UT). DEVDase activity was assessed using 10 μM Ac-DEVD-Afc as substrate. *p ≤ 0.05; **p ≤ 0.01; ***p ≤ 0.001. Additional file 2: Figure S2. NF-κB is functional in all NBL cell lines. **A**. Cells were left untreated (−) or were treated with 100 ng/ml TNFα for the indicated times. IκBα degradation was assessed by Western blot. **B**. Expression of the NF-κB -target genes cIAP2 and Bcl-2 were analyzed by Western blot in NBL cell lines left untreated (UT) or treated with 100 ng/ml TNFα for 24 h.
