# Supplementary figures and images for: TNFα sensitizes neuroblastoma cells to FasL-, cisplatin- and etoposide-induced cell death by NF-κB-mediated expression of Fas
Source: Mol Cancer. 2015 Mar 19;14:62. doi: 10.1186/s12943-015-0329-x (PMC4407790; doi:10.1186/s12943-015-0329-x)

# MYCN non-amplified

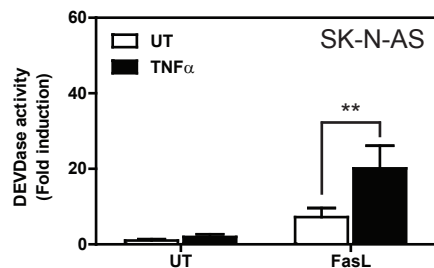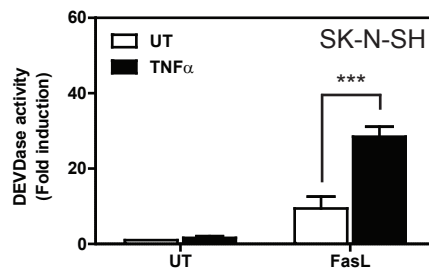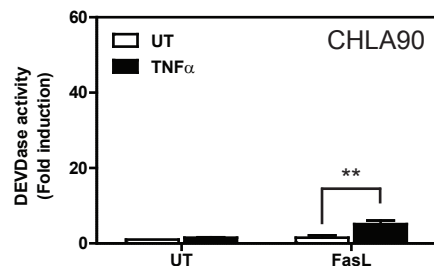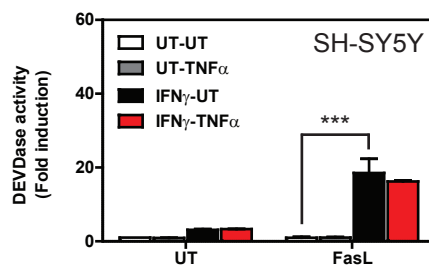

# MYCN amplified

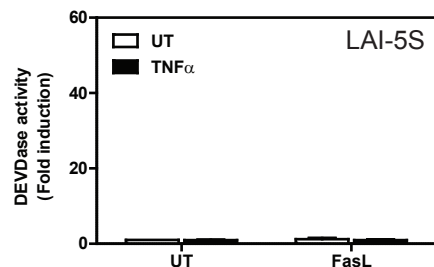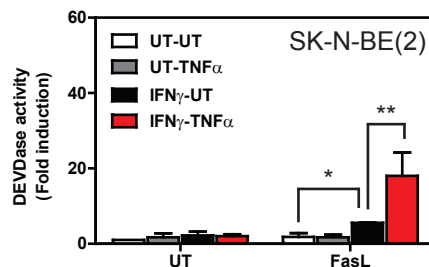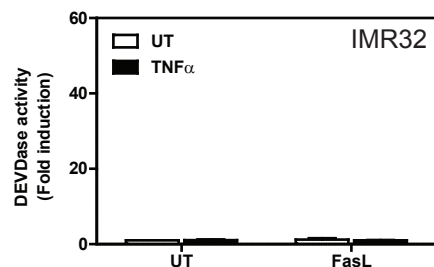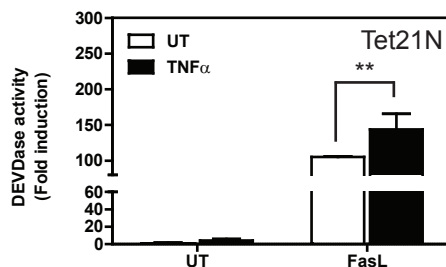

Supplement: Additional file 1: Figure S1. — TNFα pre-treatment sensitizes a subset of NBLs to FasL-induced caspase-3/7 activity. The indicated cell lines were pre-treated or not for 24 h with 100 ng/ml TNFα and were left untreated (UT) or treated for 4 h with 100 ng/ml Fc:hFasL, or 1 ng/ml Fc:hFasL for Tet21N cells. Before Fc:hFasL treatment, SH-SY5Y and SK-N-BE(2) cells were treated for 24 h with 100 ng/ml interferon-γ (IFNγ) or not (UT), and an additional 24 h with 100 ng/ml TNFα or were left untreated (UT). DEVDase activity was assessed using 10 μM Ac-DEVD-Afc as substrate. *p ≤ 0.05; **p ≤ 0.01; ***p ≤ 0.001. [file 12943_2015_329_MOESM1_ESM.pdf]

A

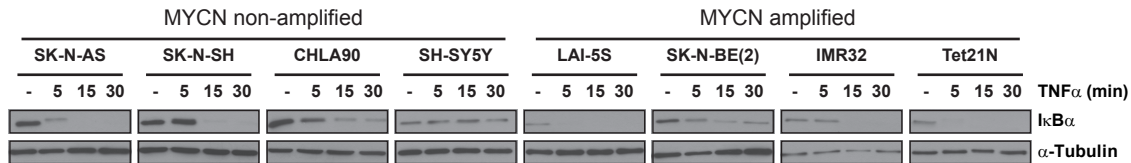

B

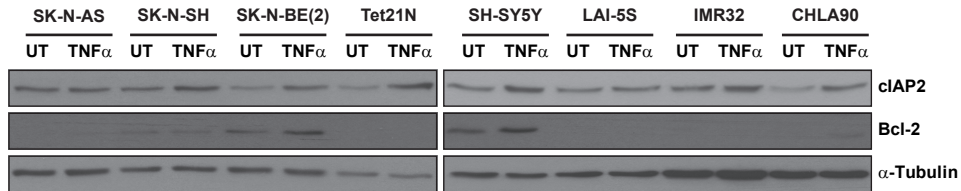

Supplement: Additional file 2: Figure S2. — NF-κB is functional in all NBL cell lines. A. Cells were left untreated (−) or were treated with 100 ng/ml TNFα for the indicated times. IκBα degradation was assessed by Western blot. B. Expression of the NF-κB -target genes cIAP2 and Bcl-2 were analyzed by Western blot in NBL cell lines left untreated (UT) or treated with 100 ng/ml TNFα for 24 h. [file 12943_2015_329_MOESM2_ESM.pdf]
